# Neurobehavioral Profiles of Six Genetically-based Rat Models of Schizophrenia-related Symptoms

**DOI:** 10.2174/1570159X21666230221093644

**Published:** 2023-07-10

**Authors:** Ignasi Oliveras, Toni Cañete, Daniel Sampedro-Viana, Cristóbal Río-Álamos, Adolf Tobeña, Maria Giuseppa Corda, Osvaldo Giorgi, Alberto Fernández-Teruel

**Affiliations:** 1 Medical Psychology Unit, Department of Psychiatry and Forensic Medicine & Institute of Neurosciences, School of Medicine, Autonomous University of Barcelona, Bellaterra, Barcelona, 08193, Spain;; 2 Department of Psychology, Austral University of Chile, Valdivia, Chile;; 3 Department of Life and Environmental Sciences (DiSVA), University of Cagliari, Sardinia, Italy

**Keywords:** Schizophrenia, selectively-bred rat models, APO-SUS, RHA, Wisket, low PPI, spontaneously hypertensive rats, brattleboro rats, shared phenotypes, prepulse inhibition, working memory, mesolimbic dopamine circuit, prefrontal cortex

## Abstract

Schizophrenia is a chronic and severe mental disorder with high heterogeneity in its symptoms clusters. The effectiveness of drug treatments for the disorder is far from satisfactory. It is widely accepted that research with valid animal models is essential if we aim at understanding its genetic/neurobiological mechanisms and finding more effective treatments. The present article presents an overview of six genetically-based (selectively-bred) rat models/strains, which exhibit neurobehavioral schizophrenia-relevant features, *i.e*., the Apomorphine-susceptible (APO-SUS) rats, the Low-prepulse inhibition rats, the Brattleboro (BRAT) rats, the Spontaneously Hypertensive rats (SHR), the Wisket rats and the Roman High-Avoidance (RHA) rats. Strikingly, all the strains display impairments in prepulse inhibition of the startle response (PPI), which remarkably, in most cases are associated with novelty-induced hyperlocomotion, deficits of social behavior, impairment of latent inhibition and cognitive flexibility, or signs of impaired prefrontal cortex (PFC) function. However, only three of the strains share PPI deficits and dopaminergic (DAergic) psychostimulant-induced hyperlocomotion (together with prefrontal cortex dysfunction in two models, the APO-SUS and RHA), which points out that alterations of the mesolimbic DAergic circuit are a schizophrenia-linked trait that not all models reproduce, but it characterizes some strains that can be valid models of schizophrenia-relevant features and drug-addiction vulnerability (and thus, dual diagnosis). We conclude by putting the research based on these genetically-selected rat models in the context of the Research Domain Criteria (RDoC) framework, suggesting that RDoC-oriented research programs using selectively-bred strains might help to accelerate progress in the various aspects of the schizophrenia-related research agenda.

## 1 INTRODUCTION

Schizophrenia is a severely disabling chronic mental disorder affecting about 1% of the world population. The main manifestations of schizophrenia are classified into ‘positive’ symptoms (*e.g*., hallucinations, delusions, thought disorders), ‘negative’ symptoms (*e.g*., social withdrawal, apathy) and ‘cognitive’ symptoms (*e.g*., attentional deficits, impairment of working memory). Antipsychotic medications are only partially effective: around 30% of patients are unresponsive to therapy [1-6].

Many human genetic studies have revealed the existence of susceptibility genes, mostly related to synaptic and glia function, neuronal growth and cortical/subcortical circuitry development (reviews by Sawa and Snyder [1]; Powell and Miyakawa [2]; González-Maeso and Sealfon [7]; Schizophrenia Working Group of the Psychiatric Genomics Consortium [8]; Flint and Munafo [9]). These studies also indicate that interactions between susceptibility genes and the environment play a prominent role [2]. Accordingly, the predominant current view is that schizophrenia is a neurodevelopmental cognitive disorder where genetic and environmental influences play a major role [1-3] that cannot be reduced to its psychotic symptoms and is not just a result of abnormal dopamine (DA) or serotonin (5-HT) functioning [10]. There is an urgent need to establish newer and heuristic animal models that can take these insights from human studies into account and show better construct and predictive validity to contribute to the development of new drugs/treatments capable of ameliorating the cognitive dysfunctions of schizophrenia. It would be of special value if the schizophrenia-relevant features/symptoms reproduced by the models were also linked to some susceptibility gene/s or neural/neurodevelopmental alterations relevant to the disorder [2, 3, 10-12].

### 1.1 Rodent Genetic Models Of Schizophrenia-relevant Features: Reverse *vs*. Forward Genetic Approaches

Recent advances in human and mouse genomics and genetic technology have led to great progress in schizophrenia research, particularly regarding its modeling in laboratory animals. Some examples of these reverse genetics models are the neuregulin 1 (*NGR1*) heterozygous mice, the transgenic mice containing inducible and/or partial *DISC1* (susceptibility gene disrupted-in-schizophrenia-1) gene mutations, the dopamine transporter knockout mice, the calcineurin (*CN*) knockout mice and the 5-HT2A and glutamate receptor knockouts [2, 3, 5, 7]. The above are examples of reverse genetics models, which start with the genes and move backward to the neurobehavioral phenotypes. These genetically-engineered rodents, as well as chemically manipulated and brain lesion-based models, are approaches of great importance because of their potential heuristic and translational value that allow the modeling of schizophrenia-relevant features from an etiology-focused (bottom-up) perspective. Notably, however, the etiological hypotheses are the main constraints of these models [2, 3, 10, 12-14]. One of their limitations stems from their frequently applied single-mechanism approach, which usually disregards many other possibly relevant neurobiological, neurodevelopmental, genetic, epigenetic, and environmental factors or processes that may be germane to the target phenotype/disease, thereby limiting their ability to identify novel important neurobiological features and targets for drug development. This is especially true when dealing with complex multifaceted and multicausal polygenic traits or syndromes, such as schizophrenia or its associated symptoms [15].

A complementary and more ethologically oriented approach would be that of utilizing top-down “behavior-to-biology” models, which adopt a forward genetic approach. These top-down approaches, rather than assuming a given pathophysiological mechanism (as do bottom-up, etiology-focused, or reverse genetics models), use a different strategy, which starts by recreating, or observing a given phenotype (*e.g*., attention deficits, impulsivity) and then follows by investigating its neuropsychological/neurogenetic underpinnings. Some of these top-down preclinical models select groups of animals from a heterogeneous (outbred) population on the basis of the different characteristics of a behavioral trait or response(s) ideally related to the target human phenotype(s) or symptoms to be modeled. Some examples are the Lister-Hooded rats selected for excessive premature (impulsive) responses in the “5-choice serial reaction time task” 5C-SRTT [13, 16-18], Wistar rats stratified for extreme values of schedule-induced polydipsia (also in the context of impulsivity; *e.g*. [19]) or genetically heterogeneous rats selected for high or low levels of prepulse inhibition (PPI) of the startle response (a phenotype related to schizophrenia; [20-22]).

Other top-down or “behavior-to-biology” models use selective bidirectional breeding for a given phenotype and then systematically evaluate the underlying neurobiological/genetic mechanisms and associated traits. Genetically-derived rat models based on selective breeding for specific phenotypic differences have received less attention than genetically-engineered rodent models. This is so despite the evident heuristic potential, and better translatability of studies using intact animals based on a forward genetics approach which, similar to the strategy used by genome-wide association studies (GWASs) of schizophrenic patients, takes advantage of the quantitative phenotypic variation to study its association with “normal” genotypic variation to find “candidate quantitative genes”. GWAS studies are consistently providing genetic and neurobiological evidence pointing to schizophrenia as a neurodevelopmental polygenic disorder whose etiology involves over a hundred genes and their interactions with the environment and ontogeny [8, 9].

The constellation of features and symptoms of schizophrenia (*e.g*., postpubertal onset, hippocampal damage, deficits of cortical functions and PPI, and limbic DA transmission dysregulation, among others) have been difficult to reproduce with a unitary animal model. Much effort has been dedicated over the last decades to the development of animal models that mimic a wide range of symptoms of schizophrenia. In this regard, there are now a few rat lines/strains that have been selectively bred based on a key phenotype.

The present article presents a non-exhaustive overview of the main phenotypic profiles of the most relevant rat models of this kind. First, we describe the phenotypic (symptom) profiles and associated neurobiological traits of these genetic rat models following the classical “diagnostic-oriented” approach, *i.e*. beginning analysis of each particular model according to the (supposedly) schizophrenia-relevant cluster of phenotypes or “symptoms” it shows, and then describing its neurobiological characteristics. Second, in section 8.1, we propose a different approach to the research with these rat models along the lines defined by the RDoC (Research Domain Criteria) framework. Rather than starting by trying to model symptoms of a diagnostic category, the RDoC approach proposes a neuroscience- and genomic-based strategy to systematically study neurobiological (molecular, cellular, circuit-level) processes influencing important behavioral phenotypes along five dimensions (cognitive system, social processes, positive emotion, negative emotion, arousal) (see section 8.1).

## 2 APO-SUS AND APO-UNSUS RATS

The apomorphine susceptible (APO-SUS) and unsusceptible (APO-UNSUS) rat lines are the result of bidirectional selective breeding of a Wistar stock of rats for extreme stereotypic responses to the nonselective D1 and D2 DA receptor agonist apomorphine [23, 24]. Cools, *et al.* hypothesized that the bimodal shape of variation in “fleeing” and “freezing” rats of an outbred strain of Wistar rats is part of an overall bimodal variation in behavioral responses to the administration of drugs that selectively alter, or reflect, the function of several neurotransmitters (*e.g*., noradrenaline, DA and GABA) in different pathways of a neural circuitry encompassing the nucleus accumbens (NAcc), the dorsal striatum, the substantia nigra pars reticulata, and the deeper layers of the superior colliculus. Hence, the “fleeing” and “freezing” rats, each one characterized by their consistency in pharmacological and behavioral responses, represent the two fundamentally different types of individuals which normally exist in unselected populations of rodents [23]. Importantly, these authors also postulated that the pharmacogenetic selection of APO-SUS and APO-UNSUS rats, *i.e*., one individual-specific feature of the overall bimodal variation for pharmacological responses in the outbred strain of Wistar rats, is a valid tool to separate the above mentioned individual-specific features as far as possible in opposite directions [23]. Moreover, a bimodal variation in the neurochemical features and functional tone of the above-mentioned circuitry, in which the NAcc plays a pivotal role, underlies the overall bimodal variation in the pharmacological and behavioral responses of APO-SUS and APO-UNSUS rats. Thus, compared with their APO-UNSUS counterparts, APO-SUS rats display higher basal locomotor activity and more robust amphetamine-induced locomotor sensitization as well as novelty-seeking behavior [23]. Moreover, APO-SUS rats self-administer cocaine more readily than APO-UNSUS rats [25]. Regarding attentional processes, APO-SUS rats show PPI deficits at low prepulse intensities and display lower levels of latent inhibition (LI) than APO-UNSUS rats [24]. It has been suggested that the PPI and attentional deficits may be due to alterations that APO-SUS rats present in central catecholaminergic nigrostriatal and limbic systems, as they exhibit enhanced DA release in the NAcc along with an increased density of DA D2 and D1 receptors [25, 26]. The finding that APO-SUS rats are more sensitive to the locomotor-stimulating and sensitization effects of some dopaminergic agonists adds experimental support to the view that the catecholaminergic activity in their limbic system is higher than that of APO-UNSUS rats [11, 25-30].

APO-SUS rats show increased metabolic activity and lower levels of dynorphin-B than APO-UNSUS rats in the hippocampus (HPC) [25]. Moreover, there are significant differences in the endocrine and immunological systems of the two lines; as compared to their APO-UNSUS counterparts, APO-SUS rats have a hyper-reactive hypothalamus-pituitary-adrenal axis [25]. Remarkably, APO-SUS rats have recently been shown to display axonal hypomyelination of GABAergic (parvalbumin) interneurons in the prefrontal cortex (PFC), which is associated with PFC dysfunction, and both processes are rescued by enriched infantile stimulation [31]. Importantly, chronic antioxidant treatment (between 5 and 90 postnatal days) with the glutathione precursor N-acetylcysteine (NAC) has been reported to restore not only antioxidant-related mRNA expression but also myelin-related mRNA expression and medial PFC-dependent cognitive alterations in the APO-SUS rat model, thus highlighting oxidative stress as a factor likely contributing to cognitive deficits in APO-SUS rats and suggesting that antioxidant treatment during neurodevelopment or prodromal stages might be useful as a therapy (or preventive treatment) in at-risk schizophrenia patients [32].

There is also evidence that other environmental and genetic factors play a role in determining the phenotypical expression of apomorphine susceptibility, as well as the pathophysiology of schizophrenia [33, 34]. Thus, cross-fostering (*i.e*., changing the dams immediately after birth) was found to reduce apomorphine susceptibility in cross-fostered APO-SUS rats reared by APO-UNSUS dams to the level of control (in-fostered) APO-UNSUS rats, whereas maternal deprivation (*i.e*., a single 24 h separation of the mother from its pups) increased apomorphine susceptibility in APO-UNSUS rats. Interestingly, cross-fostering did not affect apomorphine susceptibility in APO-UNSUS rats, and maternal deprivation did not affect apomorphine susceptibility in APO-SUS rats [33]. Therefore, the phenotypical expression of the genotype appears to be determined by the interaction between the genetic background and early environmental conditions [26, 35].

One of the strong points of the APO-SUS rats is their face validity in as much as they exhibit a wide spectrum of behaviors and neurochemical traits that resemble those of patients with schizophrenia [11, 25]. However, there is a relative paucity of information about differences between the two rat lines concerning negative and cognitive symptoms [11]. Further systematic pharmacological studies focused on the effects of atypical antipsychotic drugs and neurochemical assessments of the central serotonin and glutamate transmission are therefore warranted to obtain a more detailed picture of the validity of the APO-SUS/APO-UNSUS lines as tools to study schizophrenia-relevant symptoms and pathophysiology (Table **1**).

## 3 BRATTLEBORO RATS

The Long Evans (LE) strain-derived Brattleboro rats (BRAT) are characterized by a single mutation that impairs vasopressin release [36]. BRAT rats are therefore a valid model of diabetes insipidus [37, 38]. Regarding schizophrenia-relevant phenotypes, BRAT rats display locomotor hyperactivity and increased baseline startle response as well as an innate deficit of PPI that can be reversed by a wide range of typical and atypical antipsychotics [39-45]. De Wied *et al.* [39] reported that BRAT rats exhibit cognitive deficits, such as impaired fear conditioning, impaired acquisition and maintenance of passive avoidance behavior and deficits in spatial learning in a delayed alternation task, together with deficits in a task based on the 5-choice serial reaction time (5-CSRT) task where the BRAT rats needed 16.8% more time to complete the task than heterozygotic and Long-Evans rats. There are several reports that show that the BRAT rats also exhibit a wide range of cognitive impairments regarding reference and working memory, along with a deficit in cognitive flexibility [46-49]. Moreover, Feifel *et al.* [43] showed that BRAT rats display an innate deficit in social discrimination [45], as they spent a comparable amount of time exploring both novel or familiar rats.

Reduction in event-related potential (ERP) peak amplitudes is an endophenotype of schizophrenia that has been related to communication capacity and asociality. Remarkably also, beginning in infancy BRAT rats show deficits in ERP, suggesting an impairment of auditory information processing related to their social behavior deficits and to the reduction of vasopressin [50].

BRAT rats exhibit alterations in central serotonin and noradrenaline systems [51, 52], decreased dopamine levels in the PFC as compared to Long Evans rats [40], and dopamine D2 receptor up-regulation in the NAcc shell and dorsomedial caudate putamen [40, 53]. It has been suggested that this feature is due to a compensatory response to the low levels of DA function [40, 53].

Despite some controversial or negative findings in the cognitive domain [54, 55], it seems that BRAT rats exhibit relevant face validity, since they reproduce some phenotypes reminiscent of positive, negative and, in particular, cognitive schizophrenia-related symptoms. Another strength of the BRAT rats is their apparently high predictive validity, as their PPI deficits are reversed by some typical (haloperidol) and atypical (clozapine, risperidone, olanzapine) antipsychotics [40, 45]. On the other hand, the scarcity of data regarding neuroanatomical and functional aspects of some key neural circuits involved in reward mechanisms and in neurochemical/molecular brain processes, are eventual weaknesses of this animal model of schizophrenia-linked symptoms (Table **1**).

## 4 THE LOW- AND HIGH-PPI RAT LINES

This animal model is based on the bidirectional selective breeding of Wistar rats for high or low prepulse inhibition (PPI) of the acoustic startle response (ASR). This selection procedure has generated two rat lines that markedly differ in PPI performance across generations [56, 57]. Interestingly, these differences are present at all prepulse intensities (68, 74, 80, 86 dB). Baseline ASR levels also differ between the two lines, with the low-PPI rat line showing enhanced ASR [56, 57]. With regard to the ontogeny of those line-related differences, it is remarkable that PPI deficits in the low-PPI line can be detected already at weaning (postnatal day –PND- 21), while the increased ASR magnitude is not observed until puberty (from PND 35). On the other hand, PPI significantly increases with age in the high-PPI, but not in the low-PPI line [56, 57]. It is also noteworthy that haloperidol (0.1 mg/kg), but not clozapine, was able to reverse the PPI deficit of the low-PPI rats [58].

Freudenberg *et al.* [56] evaluated the low- and high-PPI rat lines in different spatial and operant learning paradigms to assess their learning and memory abilities as well as their behavioral flexibility. The main finding of this study was that low-PPI rats showed enhanced perseveration in both spatial and operant tasks for behavioral flexibility, while learning and memory were unaffected [56]. This is in line with the finding that perseveration is one of the most consistent traits observed in schizophrenics performing the Wisconsin Card Sorting Test (WCST), whereas there are usually no deficits in global cognitive function [59, 60]. Furthermore, Dieckmann *et al.* [61] reported that low-PPI rats display deficits in social behavior and motivation, supporting their validity as a model for some negative symptoms of schizophrenia.

Notably, although no line-related differences in DA D2 receptors have been demonstrated compared to the high-PPI line, the low-PPI line displays lower neuregulin-1 (*Ngr1* gene) methylation (suggesting increased neuregulin-1 levels) in brain regions considered to be relevant in schizophrenia, such as the medial prefrontal cortex, the nucleus accumbens, and the ventral hippocampus [62]. This finding is consistent with phenotypes found in patients with schizophrenia [63]. Moreover, Alam *et al.* [64] found that the PFC of the Low-PPI rats is hypoactive, whereas the NAcc’s activity is increased. These results are consistent with the observation that patients with schizophrenia display decreased frontocortical activity and increased activity in subcortical areas [65-67].

The face validity of this model is based on two facts: (1) the bidirectional selection of the high- and low-PPI rats has led to a clear segregation between both strains that is stable across generations, and (2) unaffected relatives of schizophrenia patients also show PPI deficits (*e.g*. [68-71] and references therein). Moreover, as mentioned above, apart from the PPI deficit, there are other cognitive/behavioral impairments that add face validity to the low-PPI rat model.

As to construct validity, however, there is a scarce amount of data in relation to the activity of some neurotransmitters that are known to play a key role in the pathophysiology of schizophrenia, such as dopamine, serotonin and glutamate. Nevertheless, the previously mentioned neurochemical/epigenetic findings suggest that rats selectively bred for low PPI may represent a potentially useful genetic model to study the pathophysiology of schizophrenia and also to evaluate new therapeutic strategies. As an example of this, electroconvulsive stimulation-therapy has been found to improve PPI in the low-PPI rat line [72]. In addition, deep brain stimulation (DBS) of the centromedian-parafascicular complex (CM-Pf) or globus pallidus internus (the entopeduncular nucleus –EPN- in rats) led to improvements in PPI in that rat line, whereas DBS of the NAcc had little effect, highlighting the likely usefulness of this rat model to investigate the pathophysiology of PPI impairment and possible treatment mechanisms (Table **1**) [73].

## 5 WISKET RATS

The Wisket rats were developed through a selective breeding program in which Wistar-derived rats reared in isolation were treated with ketamine for 15 days starting on PND 35 [74-77]. At 9 weeks of age, the rats were stratified according to their scores on three tests: the tail-flick test (pain sensitivity), PPI, and the novel object recognition (NOR) test. Rats of each sex exhibiting impaired pain sensitivity (*i.e*., long tail-flick latencies), low PPI levels and deficits in the NOR task were used for further selective breeding to generate a new rat line aimed at modeling schizophrenia-relevant symptoms [77].

In addition to selecting the rats for their scores in the three tests mentioned above, the authors tested whether selective breeding alone, namely without any further treatment, *i.e*., “Selectively bred-Untreated”, induces schizophrenia-relevant behavioral effects that are further increased by adding chronic ketamine and social isolation treatments. That is to say, are the “Selectively bred-Untreated” rats equally or less impaired than the “Selectively-bred-Treated” rats? (“Treated” denotes ketamine and social isolation treatments). The authors found that the selectively bred groups (both “Treated” and “Untreated”) displayed lower PPI levels than their non-selected —control— counterparts, while ketamine and social isolation treatments did not add any further deleterious effect. Thus, the authors argued that the genetic background (*i.e*., selective breeding) has a more relevant effect on the outcome of this test (PPI) than both ketamine and social isolation treatments [77].

Regarding the NOR test, it was found that both “Treated” groups and both “Selectively bred” groups presented impairments with respect to “Naive-Untreated” rats [77]. Besides these alterations, the Wisket rats present hyperlocomotion, decreased reference and working memory and also diminished social behavior [76, 78]. Interestingly, Horvath *et al.* [79] have reported that 10-day multidimensional (multifaceted) cognitive training rescues the motivational, attentional and learning alterations of Wisket rats [80], thus resembling the beneficial effects of cognitive-behavioral therapy in patients with schizophrenia.

Along with the above behavioral/cognitive and motivational impairments, Wisket rats show decreases in the fronto-cortical and HPC thickness, and moderately enlarged lateral ventricles [80]. As for neurochemical features, Banki *et al.* [81] have found that the mRNA levels of the oxytocin receptor are decreased in the PFC, striatum, brainstem, and olfactory bulb, as it is also observed in patients with schizophrenia [82]. Finally, some studies have found alterations in the opioid and cannabinoid systems of the Wisket rats [83, 84], and multiple alterations of D2 receptor and signaling in different brain regions have also been reported [85].

The Wisket model underscores the importance of selective breeding as a method for the assessment of complex traits influenced by genes and environmental factors. Summing up, there is at present some good evidence on the face validity of the Wisket model, which appears to be a potentially promising one to study schizophrenia-relevant features. There is, however, a paucity of information on some of the most important neurotransmitters involved in the pathophysiology of schizophrenia apart from dopamine, such as serotonin and glutamate, and the neuroanatomical, functional, and psychopharmacological profiling of this model (Table **1**).

## 6 THE SPONTANEOUS HYPERTENSIVE RAT (SHR)

Derived from Wistar Kyoto (WKY) rats, Spontaneous Hypertensive Rats (SHR) were introduced in the 1960s as a genetic model of hypertension [86]. More recently, they have been proposed as a model for Attention Deficit and Hyperactivity disorder (ADHD) and also for schizophrenia-related features [87, 88].

Regarding schizophrenia-related phenotypes, the SHR rats exhibit hyperlocomotion, deficits in social interaction, contextual fear conditioning, PPI and LI, most of them normalized by atypical antipsychotics [88-92]. Still, in the context of the predictive validity of this model, it has been reported that some deficits concerning positive and negative symptoms (*e.g*., hyperlocomotion, social interaction deficits) are improved by some cannabinoid agonists in SHR rats (Table **1**) [93, 94].

Since SHR rats are predominantly used as an ADHD model, the neurochemical studies on these rats have mainly focused on the dopaminergic system (*e.g*., [95]). Among other findings, these studies have shown that the concentration and turnover of dopamine are reduced in the mesostriatal system, whereas dopamine levels are increased in the mesolimbic pathway in SHR rats [95]. Additionally, it has been reported that the expression of the DA D4 receptor gene (*DRD4*) and DA release are decreased in the PFC of SHR rats [96, 97]. There are some contradictory results regarding DA D1 and D2 receptors; thus, some authors have found increased receptor densities in the striatum of SHR *vs*. WKY rats [98-102], while other authors found no difference between the two strains [103-105]. Additionally, Watanabe *et al.* [102] reported that the levels of the DA transporter are elevated in the striatum of SHR rats. Although these alterations of the dopaminergic pathways may explain some of the ADHD-like symptoms, such as hyperactivity, attention deficits and impulsivity, these traits of SHR rats are not attenuated by the administration of dopamine agonists [89, 90, 106, 107].

Regarding glutamate transmission, Diana *et al.* [108] found a down-regulation of *Gria1* (Glutamate ionotropic receptor AMPA type subunit 1) and *Grin1* (Glutamate Ionotropic Receptor NMDA Type Subunit 1) genes in the NAcc of SHR rats. Moreover, some authors have found altered GABAergic and cholinergic transmission in the PFC of SHR rats that could explain some of the schizophrenia-like traits of these rats [108, 109].

Altogether, the aforementioned behavioral phenotypes of SHR rats support the face validity of the model. However, there are some relevant contradictory findings, *e.g*., it has been reported that SHR rats show better spatial learning performance in the Morris water maze than Sprague-Dawley and WKY rats [110]; in addition, Buuse [111] reported no PPI deficits in SHR rats.

It is noteworthy that the majority of the authors agree that the deficits exhibited by SHR rats can be reversed by antipsychotics, supporting a high level of predictive validity.

Finally, since the assessment of the construct validity of the model has mainly focused on characteristics of dopaminergic transmission, research on other neurotransmitters and brain areas is warranted (Table **1**).

## 7 RHA (*vs.* RLA) RATS

Another genetically-based rat model for schizophrenia-relevant phenotypes is constituted by the RHA (Roman High-Avoidance) and RLA (Roman Low-Avoidance) rat strains/lines, bidirectionally (psychogenetically) selected and bred for their rapid *vs.* extremely poor (respectively) acquisition of the two-way active avoidance response [67, 112-117].

A wealth of evidence has accumulated for over 55 years indicating that the RHA strain exhibits reduced anxiety and stress-sensitivity, relative to RLAs and various outbred rat strains, in both unconditioned and conditioned tests or tasks. RHA rats generally show a pro-active coping style, and disinhibited and impulsive behavior [67, 115].

Interestingly, regarding schizophrenia-relevant phenotypes related to positive symptoms, RHA rats present increased locomotion under novelty situations and enhanced locomotor hyperactivity and/or stereotyped responses following psychostimulant or NMDA-receptor antagonist administration [115]. Importantly, in addition, RHA rats exhibit enhanced locomotor as well as mesolimbic dopaminergic sensitization to repeated (DAergic) psychostimulant or morphine administration [67].

With regard to negative-like schizophrenia symptoms, we have recently reported that, relative to RLAs and genetically heterogeneous (NIH-HS) rats, male RHA rats show lowered preference for social interaction with an unfamiliar conspecific [118, 119] (reviewed by Fernández-Teruel *et al.*, [115]). Other aspects of social behavior also differ between RHA and RLA rats since it has been shown that maternal and nesting behaviors are of poorer quality in the former strain [120] (reviewed by Fernández-Teruel *et al.*, [115]), and also that RHA rats display decreased social activity in a resident-intruder test [121].

Remarkably also, social interaction preference is much more impaired in RHA than RLA rats following the administration of dizocilpine (MK801; an NMDA-receptor antagonist), and such an impairing effect is attenuated in RHA rats by the co-administration of the atypical antipsychotics clozapine, ziprasidone and aripiprazole [115]. These antipsychotic drugs also attenuate the locomotor-stimulating effect of dizocilpine more markedly in RHA than RLA rats [115, 122].

Hence, RHA rats present a number of phenotypical traits that give support to their face and predictive validity as a model for positive- and negative-like schizophrenia symptoms.

The increased vulnerability of RHA rats to the locomotor and mesolimbic-sensitizing effects of chronic psychostimulants goes along with their enhanced preference for novelty, natural rewards and drugs of abuse. Actually, RHA rats self-administer cocaine and alcohol more readily than RLA rats by Giorgi *et al.*, [67]. This, along with the finding that RHA rats exhibit an enhanced functional tone of the mesocorticolimbic DAergic system (and several other neurochemical findings; see below) has led to the proposal of RHA rats as a valid model for research in drug addiction vulnerability and its neural basis [67, 115].

Concerning the attentional and cognitive symptoms, RHA rats show impaired acquisition and retention of fear conditioning in various paradigms (*e.g*. [123]), worsened performance in spatial reference and working memory tasks, as well as in cognitive flexibility and non-spatial learning tasks [20, 124-128].

The RHA rat line/strain has also been shown to display enhanced impulsive behavior in the 5-CSRTT, the delay discounting and DRL-20 operant tasks [128-130]. Still in the attentional/cognitive domain, it is remarkable that the RHA rat strain exhibits clear deficits (compared with RLAs and with outbred rats) of latent inhibition (LI) and prepulse inhibition (PPI) of the startle response, as well as impairment of habituation of the startle response [20, 22, 131-134]. It is worth highlighting that the PPI impairment of RHA rats is reversed by acute haloperidol (D2 receptor antagonist) and attenuated by oxytocin [122, 135], whereas clozapine is devoid of effects, although it blocks the impairing effect of dizocilpine [122]. Conversely, the PPI deficit of RHA rats is increased by apomorphine [122]. Altogether, the above profiles suggest that RHA rats may have value for modeling certain deficits of attentional and cognitive function present in schizophrenia (Table **1**).

At the neurochemical and molecular level, recent studies have shown that RHA rats present alterations of pre-/post-synaptic markers and trophic factors in the PFC and/or HPC, such as neuregulin1, homer1, synaptophysin, brain-derived neurotrophic factor (BDNF), and others, that have been linked with glutamatergic dysfunction, PFC maturation and schizophrenia [136] (reviewed by Fernandez-Teruel *et al.*, [115]). In addition, RHA rats exhibit increased DA D1 and NMDA2B receptors in the PFC, enhanced 5-HT2A receptor in PFC and HPC and a dramatic deficit of mGlu2 receptors in PFC, HPC and striatum [136-138]. These findings seem to be overall consistent with the evidence of lowered function and volume of the PFC and HPC in RHA rats [22, 134, 139, 140], as well as with an increased density of immature dendritic spines in the PFC of this strain [141]. Hence, together with their more active mesolimbic DA system, the above-mentioned molecular/synaptic marker profiles strongly suggest that RHA rats have an immature (*i.e*., adolescent-like) PFC, which seems consistent with their behavioral/cognitive alterations and with schizophrenia-relevant features [136] Fernández-Teruel *et al.* (Table **1**) [115].

It is also noteworthy that environmental conditions during postnatal development may enduringly influence some of these genetically-based phenotypic profiles. Thus, neonatal handling (NH)-stimulation (administered from PND1 to PND21) produces long-lasting improvements in PPI and cognitive performance in RHA rats, along with strain-dependent volume alterations of HPC and amygdala (AMY) [134, 140, 142], and it also increases social interaction preference more markedly in RHAs than RLA rats [118]. On the other hand, social isolation rearing (SIR) in rats is considered a model of early adversity-induced schizophrenia-like symptoms/features with face and predictive validity [143]. SIR produces profound deleterious and enduring effects in RHA rats (but not in RLA rats), as shown by marked increases in anxiety, enhanced hyperactivity, and impairments of PPI and spatial cognition [126, 144]. These findings demonstrate that environmental influences on neurodevelopmental processes may affect the development of several of the above-mentioned genetically-based schizophrenia-relevant phenotypes, while also showing an increased sensitivity of RHA (*vs*. RLA) rats to the effects of particular developmental interventions on those phenotypes.

## 8 DISCUSSION AND FUTURE AVENUES WITHIN THE RDOC FRAMEWORK

A comparative list of the main features of the selectively bred rat strains/lines exhibiting schizophrenia-relevant phenotypes that are reviewed in this article is shown in Table **1** [145-176]. Many traits are shared by two or more rat strains/ lines. For instance, all of them present PPI deficits, all except the Low-PPI line exhibit increased locomotor activity under novelty conditions, and all show decreased social behavior, with the exception of the APO-SUS line. As to other cognitive processes, the APO-SUS, SHR, RHA and BRAT models display deficits of LI, while RHA rats together with the BRAT and the Wisket strains, exhibit deficits in reference and working memory. Of note, impaired cognitive flexibility, which is considered a core symptom of schizophrenia, is observed in APO-SUS, RHA, BRAT, and Low-PPI rats, while there is no published evidence related to this trait in the Wisket and SHR lines/strains.

With regard to the important domain of impulsivity, which, as previously described, is related to the schizophrenia spectrum, it is noteworthy that only the RHA and SHR lines/ strains show consistent impulsive profiles. Impulsivity is, in turn, related to novelty/incentive-seeking, and this trait is associated with drug-seeking and schizophrenia. In this context, it is worthwhile to emphasize that, at present, only RHA and APO-SUS rats have been shown to display enhanced novelty-seeking behavior and drug self-administration. Importantly, concerning vulnerability to drug addiction and positive-like symptoms in the rat models, only the RHA, APO-SUS and SHR rats exhibit sensitized locomotor activity following chronic psychostimulant administration, and only RHA rats have been reported to display morphine-sensitized locomotor activity [66, 67, 177]. The development of enhanced psychostimulant-induced locomotor sensitization is a crucial aspect of a model of schizophrenia since it reflects an increase in the functional tone of the mesolimbic dopaminergic system and underlies the construct and predictive validity of the model; moreover, behavioral sensitization is considered to model both the positive symptoms of schizophrenia and the vulnerability to drug abuse. Therefore, at the present time, only RHA and APO-SUS rats can be considered models of dual diagnosis schizophrenia.

It is interesting that a comparison of the six different models, which have been selected on the basis of disparate phenotypes, reveals some shared traits by all or most of the strains, such as PPI deficits (all six models), social behavior deficits (RHA, BRAT, Low-PPI, Wisket, SHR) and novelty-induced hyperlocomotion (RHA, APO-SUS, BRAT, Wisket, SHR). However, it is also noteworthy that PPI impairments are not associated with other alterations in various strains. For instance, PPI deficits are not paralleled by working memory or LI impairments in several strains, and are not associated with enhanced psychostimulant sensitization also in three of the models (Table **1**). Hence, research on these six genetic models may have the potential to improve our understanding of the neurogenetic/neurobiological mechanisms that are related to clusters of symptoms/traits relevant to schizophrenia and, on the other hand, they might also be of great utility to discern why and through what mechanisms some disease-relevant phenotypes do not cluster, in a manner similar to the heterogeneity of symptoms in patients with schizophrenia.

### 8.1 Research with Selectively-bred Rat Models of Schizophrenia and the RDoC Program

There is wide agreement that animal models recapitulating phenotypes/traits related to some of the positive, negative and/or cognitive symptoms of schizophrenia are instrumental to improving knowledge of the genetic, molecular, cellular and circuit-level mechanisms underlying these symptoms, as well as to open avenues for novel and more effective treatments [1-6, 11]. However, the existing descriptive diagnostic systems (DSM, IC10), based on symptoms and symptom clusters and designed without an accurate understanding of their pathophysiology, have shown to be limited in the long run, and the scarce knowledge of neuro-psychological mechanisms of these symptoms is an obstacle for both the development of valid animal models and novel treatments. The high degree of comorbidity among mental disorders poses extra difficulties in the development of valid and translatable animal models. For example, patients with schizophrenia have high comorbidity with anxiety- and depression-related disorders, obsessive-compulsive disorder and substance abuse disorders [178]. It should also be noted that mental disorders and diagnostic criteria have varied over the years [179, 180]. All the above factors make psychiatric evaluations subjective and the appropriate selection and effectiveness of treatments far from optimal [179, 180].

In an attempt to overcome the above issues, the Research Domain Criteria (RDoC) proposal was launched to create a framework for research stemming mainly from neuroscience and genomic research, with the aim of providing an improved scientifically-grounded basis for future classification of psychiatric symptoms/disorders and treatments [181, 182]. It attempts to describe the complete range of variation of each dimension, to gain further understanding of the normal-to-pathological continuum regarding neurobehavioral features for each dimension. The RDoC system is designed as a matrix where the rows indicate several domains or hierarchical levels of function (cognitive system, social processes, positive valence –or positive emotion-, negative valence –or negative emotion-, arousal), whereas the columns define different levels or units of analysis, going from genetic, molecular and cellular levels to the circuit level (considered the “cornerstone” of the system), and from here to the organism behavioral level.

The genetically-selected rat strains discussed here might be analyzed from the perspective of the RDoC approach. We give two examples below.

For instance, if within the “cognitive system” domain we take as the dependent variable “cognitive inflexibility” (as measured by “alternation in a T maze” and by a set-shifting task), the APO-SUS (*vs.* APO-UNSUS) rats have been reported to exhibit: (i) decreased expression of the myelin-related genes proteolipid protein (*Plp1*), myelin basic protein (*Mbp*), claudin 11 (*Cldn11*), myelin-associated OL basic protein (*Mobp*), myelin OL glycoprotein (*Mog*), and myelin-associated glycoprotein (*Mag*) in mPFC (genetic level); (ii) enhanced limbic D2 receptor binding, increased DA release in the NAcc and hypomyelination of cortical GABA interneurons (molecular level); (iii) dysfunctional PFC circuits due to reduced interneuron excitability and perhaps related to mesocorticolimbic DAergic circuit anomalies (circuit level); and, (iv) PFC-dependent cognitive alterations (cognitive inflexibility; organism level). These anomalies in interneurons and PFC function impairment are related to cognitive inflexibility in rats and are reminiscent of the interneuron, PFC and cognitive abnormalities described in schizophrenic patients (*e.g*., Maas *et al.* [31, 32], and references therein). In addition, chronic treatment (between postnatal days 5 and 90) with the antioxidant glutathione precursor N-acetylcysteine (NAC) ameliorates cortical hypomyelination and cognitive deficits, leading the authors to conclude:

“The fact that in APO-SUS rats, we found impaired glutathione metabolism already at birth that persisted into late adulthood suggests that oxidative stress in SZ (*schizophrenia*) may occur before the clinical symptoms of the disorder become apparent. (...) Collectively, the findings increase our understanding of the neurobiological mechanisms leading to cognitive symptoms in SZ (*schizophrenia*) and encourage the use of chronic NAC treatment as a preventive measure for individuals at high risk for developing SZ (*schizophrenia*) and early-phase SZ (*schizophrenia*) patients” [32].

This work with the APO-SUS rats constitutes an elegant example of an RDoC-like research program on a schizophrenia-related trait (*i.e*., cognitive inflexibility), and how progress can be made from the genetic level through the molecular, cellular and circuit levels to the behavioral phenotype (cognitive flexibility) and proposal of preventive treatment. Interestingly, a recent meta-analysis [183] concluded that NAC may be useful in patients with schizophrenia, although only as an adjunct to standard treatment.

In another example, if we consider “working memory”, latent inhibition or PPI, or drug addiction vulnerability as the dependent variables, corresponding to the “cognitive system” (working memory, latent inhibition, PPI) or “positive valence” domains (addiction proneness), the RHA (*vs*. RLA) rat strain exhibits: (i) alterations of the expression of *Grm2* (mGlu2 receptor), *BDNF*, neuregulin-1 (*Nrg1*), *Homer1*, *Grin2b* (NMDA2B receptor subunit), *Vamp1* and *Snapin* genes in PFC or HPC (genetic level); (ii) alterations of glutamate transmission (*e.g*. higher NMDA-receptor antagonist-induced hyperactivity), as well as of D1, D2, D3, NMDA2B, neuregulin-1, mGlu2, and 5-HT2A (among other) receptors or proteins in PFC or HPC (molecular level); (iii) decreased activation (measured by c-fos expression) of parvalbumin GABA interneurons and increased density of “thin” (immature) pyramidal dendritic spines and astrocytes in PFC (cellular level); (iv) increased funcional tone of the mesolimbic DAergic system, *i.e*. enhanced vulnerability to chronic psychostimulant-induced locomotor and NAcc sensitization, decreased task-induced activation of the PFC and HPC (measured by c-fos expression), and immature PFC (circuit level). Alterations of various of the above-mentioned genes, neurotransmitters, receptors and circuits have been related to impairments of working memory and PPI in rodents (as it occurs in RHAs), and to enhanced vulnerability to drug sensitization and addiction (as it occurs in RHAs), which is reminiscent of the attentional/cognitive symptoms as well as proneness to drug abuse in schizophrenia (*e.g*., [67, 115], and references therein).

Still from an RDoC perspective, the other four rat strains have accumulated somewhat less evidence at the level of genetic, molecular, cellular or circuit processes. As a non-exhaustive summary of the main findings:

1) The BRAT strain, which shows enhanced novelty-induced activity (“arousal” domain), and deficits of PPI, latent inhibition and working memory (“cognitive system” domain), exhibits decreased dopamine levels in the frontal cortex and enhanced dopamine D2 receptors in the striatum, as well as augmented 5-HT levels in the frontal cortex, hippocampus and amygdala (molecular and circuit level) (Table **1**).

2) The Low-PPI rat strain displays impaired PPI and cognitive flexibility (“cognitive system” domain), and shows hypomethylation of neuregulin-1 gene (*Nrg1;* increased expression) in PFC, NAcc and vHPC (genetic level), together with decreased or increased neuronal activity in the PFC and NAcc, respectively (circuit level) (Table **1**).

3) WISKET rats, which exhibit enhanced novelty-induced activity (“arousal“ domain), and impaired PPI and working memory (“cognitive system“ domain), show decreased mRNA level of the oxytocin receptor in the PFC and striatum (genetic level), increased D2-receptor protein levels and D2-receptor G-protein activation (molecular level) in PFC, HPC and striatum (circuit-molecular level), a decrease in the frontal cortical thickness and the hippocampal area, as well as cell disarray in the CA3 subfield of the HPC (circuit level) (Table **1**).

4) Finally, SHR rats, which display increased novelty-induced locomotion (“arousal” domain), and impaired PPI, latent inhibition and aversively-conditioned contextual memory (“cognitive system” domain), exhibit enhanced psychostimulant-induced locomotion (phar-macological/molecular level), down-regulation of *Gria1* and *Grin1* genes in the NAcc (genetic level), increased dopamine levels in the NAcc and decreased in the PFC, and enhanced density of dopamine D1 and D2 receptors in the striatum (molecular and circuit level) (Table **1**).

The above examples (particularly regarding APO-SUS and RHA rats) illustrate how a genetic- and neuroscience-based (RDoC-like) approach might lead to advances in understanding (some of) the neurogenetic and circuit mechanisms related to specific behavioral phenotypes/traits (*e.g*., cognitive flexibility, working memory, latent inhibition, PPI, drug addiction vulnerability), which in turn may be important for symptoms that are currently classified as relevant for a particular disorder, namely schizophrenia.

## CONCLUSION, LIMITATIONS AND PERSPECTIVES

The selective breeding procedures developed in rats usually produce animals that mimic several schizophrenia-relevant symptoms (which is not so common when using knockout mice). Moreover, the “normal” selection (or variation) of traits/phenotypes and gene-environment interactions in humans are better (and more realistically) mimicked by selective breeding on the basis of values of a given phenotype (which often involves the co-selection of related phenotypes, as seen in some of the rat strains reviewed here) than by knockout or transgenic mice [11].

This methodology has some limitations, such as that inbreeding (or outbreeding within closed and relatively small colonies) of these rat strains could make findings less applicable to the real human world because of their reduced genetic variation. Additionally, such a reduction of genetic variability could interfere with the identification of genes or molecular features that are involved in complex phenotypes [11].

It is also noteworthy that most of the reviewed six genetic models present striking similarities in their alterations of some behavioral phenotypes (*e.g*., PPI, social interaction, novelty-induced activity) despite they exhibit quite different neuro-molecular traits (*e.g*., Table 1, D2 receptors –RHA *vs.* the other strains-, 5-HT receptors -RHA *vs.* SHR rats-, neuregulin-1 –RHA *vs.* Low-PPI rats-). Nevertheless, there are also some strain-dependent associations between neuro-molecular processes and specific behavioral phenotypes. To cite two examples: (i) increased neuregulin-1 is paralleled by increased novelty-induced activity in RHA but not in Low-PPI rats (*i.e*. no differences in activity between Low- and High-PPI strains; [147]), and, (ii) increased density of D2 receptors in the striatum is associated with impulsivity in SHR but not in BRAT rats (Table **1**). In any case, the finding that different strain-related neuro-molecular traits are associated with (or paralleled by) common or similar behavioural phenotypes might lend support to the notion that these (similar) behavioural outcomes may arise from disparate neurobiological mechanisms. In this context, the high complexity of the many different neuro-molecular mechanisms/pathways that may be related (or contribute) to schizophrenia or its symptoms is supported by recent large-scale GWAS studies indicating that there are probably over a hundred genes involved in this disorder [8, 184, 185].

Additionally, it has to be considered that, particularly when they are used alone, most of the behavioral procedures (as listed in Table **1**) are not specific to schizophrenia-related animal models. A higher specificity of the reviewed rat models (or of some of them) might eventually be attained by assessing the effects of antipsychotic drugs on the deficits/alterations in behavioral traits and neurobiological phenotypes of the strains. This approach might ultimately give higher face, predictive and construct validity to these strains (or to some of them) as models of schizophrenia-linked features.

Let us give an example. A particular strain may exhibit alterations in PPI, latent inhibition, novelty-induced activity and psychostimulant-induced locomotor and accumbal dopaminergic sensitization, accompanied by deficits in working memory, cognitive flexibility and social behavior. Such a profile might also be accompanied by enhanced NMDA-receptor antagonist-induced hyperactivity, increased NMDA-receptor antagonist-induced deficits in PPI, working memory and social behavior, augmented vulnerability to social isolation rearing-induced deficits in some of the above behavioral traits, and altered excitatory/inhibitory balance in the PFC. If a given rat strain displays all these traits, then such a strain exhibits a cluster of phenotypes that may seem to be more closely associated with schizophrenia-linked symptoms than with other psychiatric conditions. Finally, if a number of these alterations may be specifically attenuated or reversed by typical and/or atypical (2^nd^ and 3^rd^ generation) antipsychotics (but not, let us say, by antidepressants, anxiolytics of other drugs), then such a rat strain might have good predictive validity as a model of schizophrenia-related features.

It has to be kept in mind, however, that the validity and heuristic potential (*e.g*., the capacity of generating knowledge on neurobiological mechanisms) of these animal models is provisional, as it is in dynamic interaction with the current neurobiological theories/hypotheses of the disorder, *i.e*., a current theory may add value to the validity of a particular animal model at a given moment, but findings derived from such a model may provide support to the theory/hypothesis or may challenge it. Therefore, the translatability of findings derived from these (or other) rat models to human schizophrenia-relevant aspects may be limited by the provisional nature (or temporality) of the neurobiological hypotheses, of which the dopamine hypothesis of schizophrenia is probably the most influential. The dopamine hypothesis is also controversial in some aspects (*e.g*., [186-188]). For instance, there is evidence that some patients develop sensitization to psychostimulants and that some patients using psychostimulants experience psychosis, although these are not uniform findings. On the other hand, measures of central dopamine alterations in patients with schizophrenia have led to conflicting results, which may be linked to the type of measurements made [188]. In this regard, it is noteworthy that neuroimaging studies tend to be consistent in showing enhanced presynaptic dopamine content and/or release in the striatum of patients with psychosis or schizophrenia [188-192]. Conversely, there are no consistent differences in dopamine D2 receptors (one of the main mechanisms of action of current antipsychotic drugs) between patients with schizophrenia and controls [186-190]. These are examples of inconclusive evidence in support of the dopamine hypothesis of psychosis and schizophrenia, thus highlighting the provisional nature of the hypothesis.

To sum up, from a classical “diagnostic-oriented” perspective, some of the selected rat strains we have reviewed in this article present symptoms and neurobiological alterations that make them promising tools as models for schizophrenia-relevant features. We believe that future research should especially focus on, 1) drug treatment studies to see whether these models also present good predictive validity and selectivity (*e.g*., absence of false positives or false negatives), and 2) genetic mapping and differential gene expression profiles of (selected) rats presenting extremely divergent symptoms/ phenotypes or clusters of them, in order to evaluate associations among particular genes and schizophrenia-relevant (endo)phenotypes.

Conversely, instead of a classical diagnostic-based standpoint, we may adopt a perspective of research with these rat strains oriented along the lines defined by the RDoC framework. This would imply the systematic manipulation of the independent variables (*i.e*. genetic, molecular, cellular or circuit levels) to see how this produces variation in the dependent variables, *i.e*., phenotypes related to some of the defined RDoC domains (cognitive, social, positive valence, negative valence, arousal). Such an approach should lead to a shift from the predominant correlational findings (such as many of those listed in Table **1**) towards a body of “causal” genetic- and neuroscience-grounded findings and knowledge. This novel approach might provide better specific (genetic-, molecular-, cellular- and circuit-based) knowledge on the neurobiological bases of specific phenotypes/traits that might ultimately improve both the classification of particular (mental, behavioral) symptoms and the design of more specific treatments.

## Figures and Tables

**Table 1:** Comparison of a representative sample of schizophrenia-relevant features among the six selectively-bred rat models.

-	**RHA**	**APO-SUS**	**Brattleboro**	**Low-PPI**	**“3-hit” WISKET**	**SHR**
**Behavioral Phenotypes**						
Novelty-induced locomotion	Increased [122, 145, 146]	Increased [23]	Increased [40]	No effect [147]	Increased [148]	Increased [89]
Psychostimulant-induced hyperlocomotion	Increased (acute and chronic amphetamine or cocaine) [66, 149, 150 ]	Increased (acute & sub-chronic intermittent dexamphetamine) [27-29]	-	-	-	Increased (acute methylphenidate) [151]
Sensorimotor gating (PPI)	Decreased [20, 22, 133, 134]	Decreased [24]	Decreased [40, 41, 44, 152]	Decreased [56, 61, 62, 64]	Decreased [74-77]	Decreased [94, 110]
Latent inhibition	Decreased [131, 132]	Decreased [24]	Decreased [153]	-	-	Decreased [90]
Reference memory	Decreased [126, 134]	-	Decreased [46]	-	Decreased [76]	Increased [110]
Working memory	Decreased [20, 126, 127, 142]	-	Decreased [46, 47]	-	Decreased [76]	-
Cognitive flexibility	Decreased [124, 125, 134, 154, 155]	Decreased [31, 32]	Decreased [47-49]	Decreased (enhanced perseveration) [56]	-	-
Aversive conditioned memory-fear conditioning	Decreased (cue- and context-conditioning; fear-potentiated startle) [123, 156]	-	-	-	-	Decreased (contextual fear conditioning) [90, 91]
Social behavior/ social preference	Decreased [118, 119]	-	Decreased [43, 157-160]	Decreased [61]	Decreased [78]	Decreased [89]
Impulsivity	Increased [19, 121, 128-130, 138]	-	No alteration (females) [161]	-	-	Increased [162, 163]
Novelty seeking	Increased [22, 134, 139, 164]	Increased [23]	-	-	Decreased (exploration in the hole-board and other novelty-based tests) [79, 80]	-
Drug-seeking	Increased (cocaine and alcohol self- administration) [165, 166]	Increased (cocaine self-administration) [167]	-	-	-	-
**Neurochemical Phenotypes**						
Dopamine (DA)	Increased mesolimbic functional dopaminergic tone [66, 169]	Enhanced DA release in the Nacc [25]	Decreased DA levels in FC [44]	-	-	Increased in NAcc/Decreased in PFC [97]
Dopamine D1 receptors	Increased density in limbic areas [168, 169]	-	No differences in D1 receptor binding [53]	-	-	Increased density in striatum [98-101]
Dopamine D2 receptors	Decreased density in limbic areas [164, 169]	Increased density [25]	Increased density in striatum [53]	-	Increased protein expression in PFC and HPC, and increased D2 activation of G-proteins in PFC, HPC and striatum [85]	Increased density in striatum [98-101]
Noradrenaline (NA)	Increased NA levels and release in the PFC [170]	-	Increased levels in FC, hippocampus, amygdala and hypothalamus [51, 52]	-	-	-
Serotonin (5-HT)	Increased levels in the cortex after MAO inhibition. Increased release in PFC [171, 172]	-	Increased levels in FC, hippocampus, amygdala and hypothalamus [51, 52]	-	-	-
5-HT receptors	Increased fronto-cortical [^3^H]-paroxetine and [^3^H]-citalopram receptor binding. Increased 5-HT2A receptor density in PFC [137, 138, 172, 173]	-	-	-	-	Decreased 5-HT1A receptor density in the hypothalamus [174]
Neuroregulin 1	Increased expression in PFC and increased protein levels in HPC [136]	-	-	Hypomethylation in PFC, NAcc, vHPC [62]	-	-
**Function/Volume of Cortical and Limbic Areas**						
PFC	Decreased volume, neuronal activity (c-fos), and increased density of pyramidal dendritic “thin” (immature) spines and astroglia number [22, 134, 139, 141, 144]	Axonal hypomyelination of GABAergic (parvalbumin) interneurons in PFC (associated to PFC disfunction) [31, 32]	-	Reduced neuronal activity [64]	Significant decrease in the frontal cortical thickness and the hippocampal area. Cell disarray in the CA3 subfield of hippocampus [80]	-
NAcc	No difference in volume between RHA and RLA rats [22, 134, 140]	-	-	Increased neuronal activity [64]	-	-
HPC	Decreased volume, neuronal density and function (c-fos) [22, 134, 139, 140, 144, 175]	Increased metabolic activity in the hippocampus [25]	-	-	Significant decrease in HPC thickness and cell disarray in the CA3 subfield of HPC [80]	-
AMY	Decreased volume, neuronal density and function (c-fos). [22, 134, 139, 140, 176]	-	-	-	-	-

**Note:** Similarities and differences among the phenotypes of the selectively-bred rat models, with representative references included below each described effect.

**Abbreviations:** RHA: Roman high-avoidance rats. APO-SUS: Apomorphine susceptible rats. Low-PPI: rats selectively bred for low PPI levels. SHR: Spontaneously hypertensive rats. See text for description of “3-hit” WISKET rats. NAcc: Nucleus Accumbens. AMY: Amygdala. FC: Frontal cortex. HPC: Hippocampus. MAO: Monoamine oxidase. PFC: Prefrontal cortex. vHPC: ventral hippocampus.

## LIST OF ABBREVIATIONS

ADHD: Attention Deficit and Hyperactivity Disorder
BDNF: Brain-derived Neurotrophic Factor
CN: Calcineurin
DBS: Deep Brain Stimulation
ERP: Event-related Potential
GWASs: Genome-wide Association Studies
LE: Long Evans
LI: Latent Inhibition
NAcc: Nucleus Accumbens
PFC: Prefrontal Cortex
PPI: Prepulse Inhibition
RHA: Roman High-avoidance
SHR: Spontaneously Hypertensive rats
WCST: Wisconsin Card Sorting Test
